# Evaluation of Plasticiser Levels, Phthalates and Bisphenols in Bahraini Subjects with and Without Type-2 Diabetes

**DOI:** 10.3390/jox16010015

**Published:** 2026-01-19

**Authors:** Edwina Brennan, Priya Das, Pearl Wasif, Xianyu F. Wang, Jochen F. Mueller, Chang He, Jean V. Varghese, Alexandra E. Butler, Stephen L. Atkin, Naji Alamuddin

**Affiliations:** 1 School of Medicine, Royal College of Surgeons in Ireland-Medical University of Bahrain, Busaiteen 15503, Bahrainpsulaiman@rcsi.com (P.W.); naalamuddin@rcsi.com (N.A.); 2 Queensland Alliance for Environmental Health Sciences, The University of Queensland, 20 Cornwall Street, Woolloongabba, Brisbane, QLD 4102, Australiac.he@gdut.edu.cn (C.H.); 3 Endocrinology Department, King Hamad University Hospital, Busaiteen 24343, Bahrain

**Keywords:** obesogens, type-2 diabetes (T2D), endocrine disrupting chemicals (EDCs), plasticisers, phthalate metabolites, bisphenols

## Abstract

Background: Plasticisers with endocrine-disrupting potential are ubiquitous and associate with obesity and type-2 diabetes (T2D), with higher levels reported in the Middle East. However, no data exist on plasticiser exposure in Bahrain where T2D affects 15% of the national population. Methods: An observational exploratory study in T2D (*n* = 60) and controls (*n* = 96), analysed for 24 h urinary plasticiser levels (liquid chromatography tandem mass spectrometry (LC-MS/MS)). Correlation and generalised linear model (GLM) analyses were employed to examine associations. Results: T2D were older (*p* < 0.001), had higher body mass index (BMI) (*p* < 0.001), body weight (*p* < 0.001) and glycosylated haemoglobin A1c (HbA1c) (*p* < 0.001). Correlation analysis revealed differences in inter-plasticiser, and plasticiser and biomarker relationships, with loss or reversal in T2D compared to controls. Mono-*n*-butyl phthalate (MnBP) levels were higher in T2D (*p* = 0.04); however, regression analysis revealed significant association with age. The GLM analyses demonstrated marked differences in the levels of mono(3-carboxypropyl) phthalate (MCPP), mono(2-ethyl-5-carboxypentyl) phthalate (MECPP), monoethyl phthalate (MEP) and bisphenol S (BPS), with lower levels in T2D versus controls (B = −3.41, *p* = 0.01; B = −5.28, *p* < 0.001; B = −8.94, *p* < 0.001; B = −6.09, *p* = 0.006, respectively); however, these contrasts appeared to be substantially confounded by BMI and/or age. Positive influence of age and negative influence of BMI when observed across the full dataset were generally reversed in T2D. Levels were complementary to those previously reported for the Middle East. Conclusions: The study indicates the phthalate levels in Bahrain are elevated though complementary to studies of phthalates in the Middle East; within those levels, the study indicates differential exposure–response relationships with plasticisers, influenced by age and BMI, in those with T2D compared to healthy controls.

## 1. Introduction

Type-2 diabetes (T2D) is a global health concern accounting for 95% of all diabetes cases [1]. By 2050, it is projected that more than 1.31 billion people will have diabetes, with prevalence rates as high as 16.8% in the Middle East [1]. T2D is a complex metabolic condition caused by a combination of factors including insulin resistance, physical inactivity and obesity. Genetics and demographic factors further contribute to an individual’s predisposition to the condition. More recently, it has been hypothesised that environmental pollutants with endocrine-disruptor potential may play a role in the obesity epidemic contributing to T2D [2].

Phthalates, diesters of phthalic acid (1,2-benzenedicarboxylic acid) and bisphenols are used in plastics due to their softening and hardening capabilities, respectively. Not being chemically bound to polymers, phthalate exposure results from ingestion of contaminated food stuff or through dermal contact [3]. Similarly, bisphenol A (BPA) exposure predominantly occurs through ingestion when residual monomers from manufacturing diffuse, or chemically bound monomers undergo hydrolysis [4]. Although restrictions on the use of certain phthalates exist (Commission Regulation (EU) No 10/2011), and a recent ban on BPA has been implemented (Commission Regulation (EU) 2024/3190), these plasticisers are widespread in the environment and are frequently detected in body fluids and human tissues [3,5,6]. Moreover, while suggested safe alternatives for BPA exist, bisphenol S (BPS) has been found to be as hormonally active [7] and having equivalent obesogenic effects [8].

Plasticisers have relatively short elimination half-lives in the body [9,10]. Metabolites of di-2-ethylhexylphthalate [DEHP] and di-isononylphthalate (DINP) have reported half-lives of 4–8 h following oral exposure, with 90% of metabolites present in urine after 24 h [11]. BPA has a urinary elimination half-life of 5.4 h [12]. Although plasticisers are non-persistent chemicals and do not bioaccumulate, continuous exposure can lead to high concentration levels in the body.

These chemical obesogens have the potential to impact energy balance by altering lipid homeostasis to promote adipogenesis and lipid accumulation [13]. The proposed mechanisms include the following: androgenic/anti-androgenic effects [14], disruption of the hypothalamic–pituitary–adrenal/–thyroid axis, alterations in nuclear hormone receptor (NHR) signalling [15,16] and induction of inflammation and oxidative stress [17]. Several studies provide support for risk of insulin resistance and T2D with specific plasticisers [18,19,20]. More recent meta-analysis and umbrella studies further support associations between plasticisers with obesity, T2D and insulin resistance in adults [21,22,23,24]. However, there remains inconsistency in the literature with regard to specific plasticisers and whether previously determined associations are in fact reflected in those with T2D.

Human biomonitoring studies commonly assess exposure by measuring urinary concentrations of phthalate monoester metabolites (e.g., mono-*n*-butyl phthalate [MnBP], monoethyl phthalate [MEP], metabolites of DEHP) which reflect recent exposure. Large temporal and geographic variations exist and, following regulatory restrictions of major phthalates in North America and Europe, urinary concentrations of many high-molecular-weight phthalate metabolites have declined over the past decade in those regions [25]. Whilst few biomonitoring studies exist for many low- and middle-income regions, including the Middle East, in a systematic review and meta-analysis of 216 studies spanning non-US/Canada/Europe populations, phthalates exhibited up to a ten-fold higher urinary concentration in the Middle East (and South Asia) compared with other regions [26].

The aim of this exploratory study was therefore to compare the levels of proposed obesogenic plasticisers between individuals with T2D and healthy controls. Specifically, 24 h urinary plasticiser levels were examined for twenty plasticisers, including phthalate monoesters, as measures of their diester counterparts, and bisphenols. We hypothesised that plasticiser levels would be higher in those with T2D and that patterns of association between plasticiser levels and age and body mass index (BMI) would differ between T2D and controls.

## 2. Materials and Methods

### 2.1. Study Design

The study design is a single centred observational case–control study that was conducted in a tertiary hospital in Bahrain, the King Hamad University Hospital. The study period was from 30 March to 10 April 2021. Study participants were recruited using convenience sampling by advert in the hospital and in diabetes clinics. Specifically, participants with T2D were recruited from the endocrinology outpatient clinic during routine follow-up visits. Non-diabetes healthy controls were similarly recruited from adults attending the same hospital outpatient clinics for routine check-ups. For both groups, 24 h pooled urine samples were collected at home using pre-labelled collection containers following written instruction and then returned to the hospital at the subsequent clinic visit. We did not recruit individuals who were admitted to inpatient wards, thereby minimising the likelihood that short-term hospitalisation-related exposure to plasticisers (e.g., via PVC medical devices or infusion systems) contributed to urinary levels during the sampling period. At the clinic visit, participants gave non-fasting routine clinical blood samples and anthropometric measurements were recorded. Blood and urine samples were stored at −80 °C prior to analysis.

Inclusion criteria for those with T2D included the World Health Organisation (WHO) guidelines for the diagnosis of diabetes [27] (glycosylated haemoglobin A1c (HbA1c) ≥ 6.5%, or 2 fasting plasma glucose readings of >7.0 mmolL^−1^ or 2 random plasma glucose readings >11 mmolL^−1^ in the absence of symptoms or concurrent illness or medication (e.g., thiazide diuretics) which might lead to hyperglycaemia or one reading meeting the diagnostic level in the presence of symptoms of polyuria, polydipsia, nocturia, fatigue or blurring of vision, or an oral glucose tolerance test (OGTT) using a 75 g glucose load), and being on stable medication for their diabetes, hypertension, lipids and gout, where applicable, for 3 months prior to entry into the study. Inclusion criteria for non-diabetes healthy controls included the absence of any chronic illness. Inclusion criteria for both T2D patients and non-diabetes healthy controls included >21 years of age. Exclusion criteria for both cohorts were those currently taking or who had taken antibiotics in the last 3 months and those enrolled in clinical trials. All participants gave their written informed consent to take part in the study. The study was conducted in accordance with the Declaration of Helsinki and ethical approval was obtained from King Hamad University Hospital (number 21-406), and the Royal College of Surgeons in Ireland, Medical University of Bahrain (number 210321).

### 2.2. Plasma Biochemical Measurement

HbA1c was measured using a hemoglobin testing system (VARIANT II TURBO Bio-Rad Laboratroties, Inc., Hercules, CA, USA), using the manufacturer’s recommended protocol.

### 2.3. Plasticiser Measurement

Urine sample preparation and plasticiser analysis: All glassware was washed and then baked in a muffle furnace at a temperature of 500 °C to decontaminate possible plastic-related chemicals. Consumables that cannot be furnaced were instead rinsed with a minimum of 1× liquid chromatography (LC)-grade *n*-hexane, 3× LC-grade acetone, 3× LC-gradient grade methanol, and 3× high-performance liquid chromatography (HPLC)-grade water. Briefly, urine samples from T2D patients and health controls were defrosted and mixed well before 50 µL was aliquoted into a 2 mL amber vial. Subsequently, each sample vial was spiked with 40 µL of ^13^C labelled bisphenol and phthalate metabolite mix (25 ppb in methanol). Lastly, 485 µL of ultra-pure water and 25 µL of β-glucuronidase (*Escherichia coli*-K12) solution (of at least 3.5 units of specific activity) were added to each sample vial. Samples were then incubated at 37 °C for 1.5 h before 400 µL of acetic acid (Merck, Darmstadt, Germany) solution (0.5% in water) was added. After centrifuge at 4800 rcf for 20 min, the samples were ready for instrument analysis.

Specific gravity: The specific gravity (SG) measurement for the urine samples was conducted using a digital refractometer (UG-α; ATAGO Co., Ltd., Tokyo, Japan) which was calibrated using MilliQ water before each use.

Instrument analysis: Samples were analysed using a SCIEX Triple Quad™ 7500 liquid chromatography coupled with a tandem mass spectrometer (LC-MS/MS) System—QTRAP^®^ Ready (SCIEX, Framingham, MA, USA). A Kinetex^®^ Biphenyl column (1.7 µm) (Phenomenex, Torrance, CA, USA) was used for the analysis. Oven temperature was kept at 45 °C. Mobile phases for phthalate metabolites consisting of 1% MeOH (Merck, Darmstadt, Germany) and 0.1% acetic acid in MilliQ water (solvent A) and 99% MeOH and 0.1% acetic acid in MilliQ water (solvent B) were used. Mobile phases for bisphenols were similar, without the addition of acetic acid. The mass spectrometer was operated with scheduled multiple reaction monitoring (sMRM). Two ion transitions were monitored for each target analyte and internal standard. A total of 13 phthalate metabolites were targeted, including MEP, MnBP, mono(3-carboxypropyl) phthalate (MCPP), mono(2-ethylhexyl) phthalate (MEHP), mono(2-ethyl-5-carboxypentyl) phthalate (MECPP), mono(2-ethyl-5-hydroxyhexyl) phthalate (MEHHP), mono(2-ethyl-5-oxohexyl) phthalate (MEOHP), monobenzyl phthalate (MBzP), mono-iso-butyl phthalate (MiBP), monomethyl phthalate (MMP), mono-iso-nonyl phthalate (MiNP), mono-*n*-octyl phthalate (MnOP) and monocyclohexyl phthalate (MCHP). A further total of seven bisphenols were targeted, including BPA, BP-AF, BP-AP, BPB, BPF, BPS and BPZ. All measurements were corrected for SG prior to further statistical analysis.

### 2.4. Statistics

This pilot exploratory study empirically aimed to recruit 40 T2D patients and 40 healthy controls. The power and sample size for this pilot study was based on the work of Birkett and Day [28], who concluded that a minimum of 20 degrees-of-freedom is required to estimate effect size and variability. Descriptive statistics were applied to summarise the data, with frequencies presented for categorical variables and means ± standard deviation (SD) or interquartile range (IQR) for continuous variables. The normality of plasticiser level distribution was assessed using the Kolmogorov–Smirnov test. Values below the limit of detection were imputed using multiple imputations based on a log-normal distribution [29]. Due to non-normal distribution of the data, continuous variables across groups were examined using non-parametric tests (Mann–Whitney U test). Bivariate associations between continuous measures were evaluated using Spearman’s correlation. Distribution of the plasticisers was positively right-skewed; hence, the differences between groups (T2D versus healthy controls) were assessed using generalised linear models (GLMs) with a gamma distribution and log link, adjusting for age and BMI. Group (T2D versus healthy controls) was included as a categorical predictor, with age and BMI as continuous covariates. As T2D was included as a binary variable in the model and the cohorts were age-matched, HbA1c and age were not included as continuous variables to avoid redundancy and unnecessary variability. All the tests were two-tailed and *p*-value < 0.05 was considered statistically significant. The data were subjected to statistical analysis using R studio (2023.06.2-561) and SPSS software (v.31.0).

## 3. Results

### 3.1. Demographics

60 T2D patients and 96 healthy controls were recruited to the study (Table 1). T2D patients were older (53 versus 33.6 years, *p* < 0.001) and had higher BMI (36 versus 25 kg/m^2^, *p* < 0.001) and HbA1c values (8.3 versus 5.5%, *p* < 0.001) compared to healthy controls (Table 1). Gender ratio did not differ between T2D patients and healthy controls (*p* > 0.05).

### 3.2. Plasticiser Levels

The majority (94.2–100.0%) of values for eight plasticisers (MCHP, MiNP, MnOP, MiDP, BP-AF, BPB, BPF, BPZ, BP-AP) were below the limit of detection. Therefore, we focused on the remaining twelve plasticisers: MBzP, MCPP, MECPP, MEHHP, MEHP, MEOHP, MEP, MiBP, MMP, MnBP, BPA and BPS. T2D had significantly higher median or 50th percentile levels of MnBP (16.5 versus 6.45, *p* = 0.04). MEP had the highest median levels (Table 2) but did not differ between T2D and healthy controls (*p* > 0.05), nor did the remaining ten plasticisers.

### 3.3. Correlation Analysis

Spearman’s rank order correlations of plasticisers were examined with age, BMI and HbA1c (Table 3). MBzP moderately correlated with age in both cohorts (controls: ρ = 0.348, *p* = 0.04; T2D: ρ = 0.413, *p* = 0.03). MEHHP, MEOHP, MiBP, MnBP and BPS positive correlation with age in controls (ρ = 0.277, *p* = 0.01; ρ = 0.307, *p* = 0.01; ρ = 0.296, *p* = 0.02; ρ = 0.224, *p* = 0.04; ρ = 0.249, *p* = 0.047, respectively) was reversed in the T2D group (ρ = −0.339, *p* = 0.01; ρ = −0.088, *p* = 0.59; ρ = −0.206, *p* = 0.16; ρ = −0.384, *p* = 0.002; ρ = −0.186, *p* = 0.76, respectively). MBzP positively correlated with BMI in controls (ρ = 0.253, *p* = 0.16), with the reverse relationship observed in the T2D cohort (ρ = −0.453, *p* = 0.02). MEP negatively correlated with BMI in controls (ρ = −0.317, *p* = 0.003), with the reverse relationship observed in the T2D cohort (ρ = 0.329, *p* = 0.01). Additional positive correlations were observed for BMI with MCPP and MECPP in the T2D group (ρ = 0.361, *p* = 0.03; ρ = 0.284, *p* = 0.03, respectively). In the control group, MEP and MMP negatively correlated with HbA1c (ρ = −0.235, *p* = 0.03; ρ = −0.213, *p* = 0.03, respectively) while no such correlations were observed in the T2D cohort.

**Figure 1:**
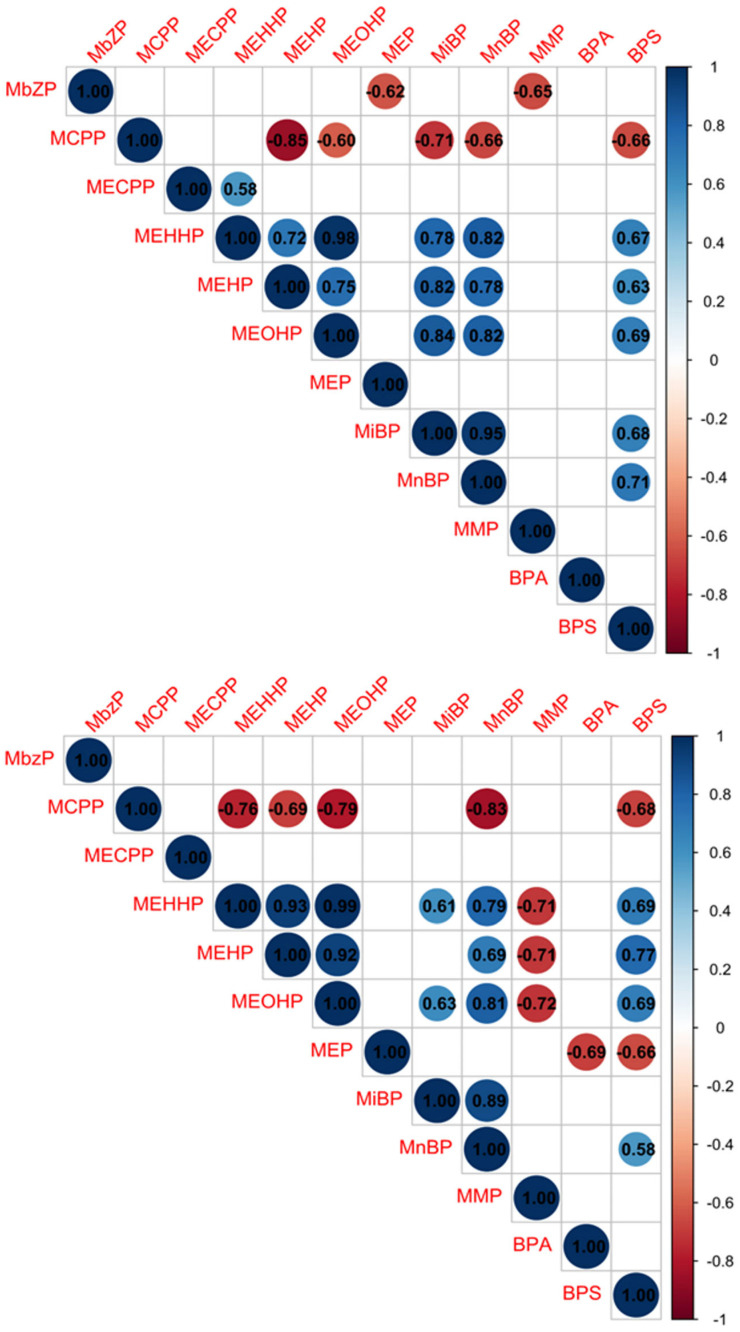
Spearman’s rank order correlation matrices of urinary plasticiser levels in their respective cohorts. In healthy controls (upper matrix) and in T2D (lower matrix). MBzP, monobenzyl phthalate; MCPP, mono(3-carboxypropyl) phthalate; MECPP, mono(2-ethyl-5-carboxypentyl) phthalate; MEHHP, mono(2-ethyl-5-hydroxyhexyl) phthalate; MEHP, mono(2-ethylhexyl) phthalate; MEOHP, mono(2-ethyl-5-oxohexyl) phthalate; MEP, monoethyl phthalate; MiBP, mono-iso-butyl phthalate; MMP, monomethyl phthalate; MnBP, mono-*n*-butyl phthalate; BPA, bisphenol A; BPS, bisphenol S; *p* < 0.05.

We also examined Spearman’s rank order correlations of plasticisers within their respective cohorts (Figure 1). While most of the correlations between plasticisers were similar in controls and the T2D group, there were also differences in their correlation patterns. In controls alone, MBzP negatively correlated with MEP and MMP (ρ > −0.60, *p* < 0.05), and MECPP positively correlated with MEHHP (ρ = 0.58, *p* = 0.049). In T2D alone, MEHHP, MEHP and MEOHP negatively correlated with MMP (ρ > −0.70, *p* < 0.05) and MEP negatively correlated with BPA and BPS (ρ > −0.65, *p* < 0.05). Positive correlations between MEHP and MiBP (ρ = 0.82, *p* = 0.0001), and MiBP and BPS (ρ = 0.68, *p* = 0.015) in controls, were lost in the T2D group.

### 3.4. Regression Analysis

GLMs showed strong separation in MCPP, MECPP, MEP and BPS levels between T2D and control groups (B = −3.41, *p* = 0.01; B = −5.28, *p* < 0.001; B = −8.94, *p* < 0.001; B = −6.09, *p* = 0.01, respectively), with T2D having lower levels than controls (Table 4). When age and BMI were accounted for, models indicated no significant difference in levels of MBzP, MEHP, MEOHP, MiBP, MnBP, MMP and BPA between T2D and controls.

A significant main effect of BMI was observed across the full dataset for MECPP, MEP and BPS levels (B = −0.1, *p* = 0.01; B = −0.26, *p* < 0.001; B = −0.36, *p* < 0.001, respectively), indicating a negative association with their levels. A highly significant group × BMI interaction was observed for MECPP, MEP and BPS (B = 0.14, *p* < 0.001; B = 0.32, *p* < 0.001; B = 0.34, *p* < 0.001, respectively), indicating that the effect of BMI on their levels reversed direction between groups.

Positive association with age for MEHHP, MEP, MnBP and BPS (B = 0.02, *p* = 0.07; B = 0.04, *p* = 0.01; B = 0.04, *p* = 0.03; B = 0.08, *p* < 0.001, respectively) in the whole dataset flips negative in T2D, thereby showing significant differences in age interaction (B = −0.06, *p* = 0.01; B = −0.02, *p* = 0.48; B = −0.06, *p* = 0.011; B = −0.09, *p* = 0.003, respectively). The model showed that the group and age interaction term for MCPP was significant (B = 0.03, *p* = 0.04), indicating that association between age and MCPP levels differ significantly between T2D and the healthy control group. GLMs for MBzP, MEHP, MEOHP, MiBP, MMP and BPA showed that none of the predictors had any interaction effects on their levels (Table 4).

## 4. Discussion

This study evaluated 24 h pooled urinary plasticiser levels in a Bahraini cohort with and without T2D. We observed several differences in inter-plasticiser and plasticiser–biomarker relationships, suggesting differing exposure–response dynamics in those with T2D compared to healthy controls. Of the twelve plasticisers examined, MnBP levels were significantly higher in the T2D cohort, yet this was attenuated when adjusted for age in regression analysis. The GLM analyses demonstrated marked differences in the levels of MCPP, MECPP, MEP and BPS, with lower levels in T2D versus controls; however, these contrasts appeared to be substantially confounded by BMI and/or age. The positive influence of age and the negative influence of BMI when observed across the full dataset were generally reversed in T2D.

MnBP is the major metabolite of dibutyl phthalate (DBP), a low molecular weight phthalate widely used in PVC products such as personal care, cosmetics and food contact materials [30,31]. DBP has been shown to migrate from PVC packaging [32], contributing to dietary [33] and topical exposure [34]. Toxicological concerns have led to restrictions in use to 0.1% in consumer products and 0.3 mg/kg in repeated use food packaging (Commission Regulation (EU) No 10/2011). Bahrain regulates the use of plasticisers mainly through Gulf Standards Organisation (GSO) standards [35], established by the GCC in 2004 to ensure the safety of products and health of its members. GSO standards, which generally align with international standards, are adopted by Bahrain Standards and Metrology Directorate (BSMD) [36], having the same restrictions on phthalates as the EU. Despite regulatory restrictions, a study revealed an exponential increase in MnBP levels globally [26]. Several mechanistic studies provide support for the obesogenic potential of DBP and its metabolite. MnBP has been shown to associate with perturbed peroxisome proliferator-activated receptor (PPAR) signalling genes [37] and to activate PPARγ [38], a regulator of adipogenesis and insulin sensitivity [39]. While DBP is anti-androgenic at high dose, hydrolysis to MnBP at low dose shifts to androgenic activity [14], potentially promoting obesogenic pathways. DBP and MnBP possess thyroid receptor antagonist activity [40] and associate with thyroid hormones [41,42], suggesting the potential to impact lipolysis and gluconeogenesis. In human adipose tissue, serum MnBP levels positively associated with biomarkers of oxidative stress [43] and inflammation [44], which are implicated in pancreatic β cell function.

In this study, we found that MnBP levels were higher in T2D versus controls, influenced by differences in age rather than being independently associated with T2D status. Consistent with this study, others report no association between MnBP and IR indices, namely glucose, HOMA-IR and insulin [18,45], nor BMI or dyslipidaemia [46]. However, there are conflicting results in the literature, with studies reporting association with increased odds of diabetes [18], HOMA-β [19] and HOMA-IR [20]. More recent meta-analyses provide strong support for MnBP’s association with risk of diabetes (OR = 1.27, 95% CI: 1.03–1.56) [24] and metabolic syndrome (OR = 1.0, 95% CI: 0.9–1.12) [47]. Discrepancies in the literature may be due to racial differences, given that phthalate dose–response relations may differ based on race and ethnicity [48], as well as differences in gender [49], plasticiser exposure profiles [5] and genetic predisposition to obesity and T2D [50].

The higher levels of MnBP in T2D, although influenced by age in this cohort, are consistent with those found in other regional case–control studies conducted in T2D populations [51,52]. Although not statistically significant, our GLM analyses indicate lower levels of MCPP, MECPP, MEP and BPS in T2D compared to controls. This contrasts with published studies where plasticisers are generally reported at higher levels in T2D versus controls. For example, there are increased levels of MEHHP, MEOHP, MECCP and MBzP [53], MEP and MEOHP [52], MECPP, MEHHP and MEOHP [54] and MBzP, MCPP, MECPP, MEHHP, MEHP, MEOHP, MEP, MiBP and MMP [51] and BPS [55]. The inconsistencies in the results here compared to other published studies may be due to differences in plasticiser measurement and adjustment. In this study, we examined a pooled 24 h urinary collection, whereas most studies used a single measurement which may not accurately assess exposure given their relatively short half-lives [2]. In addition, we used SG correction, whereas most studies used creatinine adjustment that may interfere when glucuronides or sulphate phthalate conjugates are formed [56], and are more likely to be impacted by potential confounders [57]. Although the lower levels of MCPP, MECPP, MEP and BPS observed in this study in T2D may reflect metabolic status, reverse causation cannot be excluded; disease-related behavioural changes, including alterations in diet (fresh versus pre-packaged foods), may also contribute.

T2D diabetes is a complex metabolic condition with multiple contributing factors, including higher BMI and older age [58]. While there is consensus in the literature that BMI and age also impact plasticiser levels, there are inconsistencies with regard to specific plasticisers and their directions of association [59,60]. In this study, several of the plasticisers were confounded by BMI and age. An interesting and novel finding was the reversal of these statistically significant associations in GLM analyses in the entire study cohort compared to those in the T2D group alone; MEHHP, MEP, MnBP and BPS levels negatively associated with age, and MECPP, MEP and BPS levels positively associated with BMI in T2D. This suggests altered exposure–response dynamics in T2D; however, whether these differences are a result of metabolic changes in those with T2D requires further investigation.

Compared to other regional biomonitoring studies, median plasticiser levels in this study were similar to those reported in an adult population in Iran [61], but generally lower compared to a 2006–2007 Kuwait population, 391 ng/mL MEP, 94.1 ng/mL MnBP, 67.7 ng/mL MECPP and 49.4 ng/mL MiBP [62], and a 2019–2020 Saudi population, 245.6 ng/mL MEP, 114.3 ng/mL MnBP, 39.6 ng/mL MiBP and 19.8 ng/mL MECPP [63]. Of the 12 plasticisers examined, MEP had the highest median levels in both groups, consistent with human biomonitoring studies and higher reported MEP levels in Gulf Cooperation Council (GCC) countries [5,26].

To our knowledge, this is the first study to examine plasticiser levels in a Bahraini population. The prevalence of T2D among Bahraini nationals is 15% [64] and, according to the International Diabetes Federation, Bahrain is ranked within the top 10 countries with the highest prevalence of T2D globally. Therefore, the strength of this study lies in the homogeneous study population to account for ethnicity, the measurement of several plasticisers with endocrine-disrupting and obesogenic potential, the inclusion of a potentially vulnerable group, T2D subjects and the use of 24 h pooled urine samples.

The limitations of the study include the small sample size, although power calculation was sufficient to identify differences between groups. As the study was exploratory, no formal correction for multiple testing was employed, and so the results should be interpreted cautiously. Due to the cross-sectional design and lack of data on potential exposure sources, the study could not characterise exposure duration or directly attribute plasticiser exposure to specific sources. Due to their low pH tolerance, phthalates are commonly used in coatings of enteric-coated medications to prevent drug degradation, protect against gastric irritation and support targeted intestinal drug delivery. Therefore, plastic packing of pharmaceuticals as well as pharmaceuticals themselves may pose as a potential source of exposure. While hazard index indicated no risk, the highest total levels of phthalates have been reported for cholesterol and blood pressure-lowering drugs [65]. Consequently, while regular medication use was recorded from outpatient clinic files, the plasticiser content of specific drug formulations and delivery systems was not systematically assessed, and so a contribution of medication-related exposure cannot be completely excluded. Furthermore, anti-diabetic medications may impact renal function and metabolic pathways; SGLT2 inhibitors reduce the glomerular filtration rate (GFR), easing tubular metabolic demand via improved cortical oxidation and mitochondrial function [66], while metformin activates AMP-activated protein kinase signalling and autophagy, providing renoprotective effects [67]. Such effects have the potential to impact plasticiser clearance which were not accounted for in this study.

## 5. Conclusions

The study indicates the phthalate levels in Bahrain are elevated, though complementary to studies of phthalates in the Middle East. Within these exposure levels, the study indicates differential exposure–response relationships with plasticisers, influenced by age and BMI, in those with T2D compared to healthy controls. Future studies incorporating longitudinal design and detailed behavioural and dietary assessment are warranted.

## Figures and Tables

**Table 1:** Demographic characteristics of healthy participants and T2D patients.

	Control (*n* = 96)	T2D (*n* = 60)	*p*-Value
Gender (%)			
Male	57 (59.3%)	36 (60.0%)	1.0
Female	39 (40.6%)	24 (40.0%)	
Age (Mean ± SD)	33.61 ± 8.34	52.80 ± 11.07	<0.001
BMI (Mean ± SD)	25.12 ± 2.94	36.17 ± 8.08	<0.001
HbA1c (%) (Mean ± SD)	5.48 ± 0.35	8.31 ± 1.17	<0.001

T2D, type-2 diabetes; BMI, body mass index; HbA1c, glycosylated haemoglobin A1c.

**Table 2:** Distribution of plasticisers in T2D patients and healthy participants.

Plasticiser (ng/mL)	Group	N < LOD	Min	Percentile 25th	Percentile 50th	Percentile 75th	Max	*p*-Value
MBzP	Control	62	0.035	0.22	0.43	1.08	3.80	0.74
	T2D	33	0.032	0.20	0.37	0.70	2.10	
MCPP	Control	27	0.015	0.8	1.2	1.7	11	0.61
	T2D	11	0.014	0.46	1.1	2.7	26	
MECPP	Control	-	0.25	3.425	5.85	13.25	211	0.82
	T2D	-	1.2	3.625	5.5	10	54	
MEHHP	Control	8	0.081	0.615	1.35	7.025	53	0.19
	T2D	1	0.086	0.825	3.8	8.85	69	
MEHP	Control	4	0.26	1.3	2.8	4.8	59	0.45
	T2D	-	0.51	1.4	2.1	4.7	32	
MEOHP	Control	17	0.035	0.28	0.67	4.6	25	0.36
	T2D	4	0.04	0.42	1.9	5.15	34	
MEP	Control	-	8.5	55.75	131.5	239	5109	0.75
	T2D	-	4.1	54	111	284.25	4272	
MiBP	Control	21	0.34	2.55	5	22.5	124	0.17
	T2D	11	0.37	5.1	9.1	19	90	
MnBP	Control	-	0.85	3.775	6.45	55	327	0.04
	T2D	-	1.1	5.4	16.5	44.25	242	
MMP	Control	32	0.11	2.3	3.5	5.525	33	0.42
	T2D	26	0.12	2.5	3.95	6.725	88	
BPA	Control	59	0.023	0.93	1.5	3.3	11	0.34
	T2D	27	0.026	0.9	1.3	2.4	6	
BPS	Control	25	0.04	0.048	0.12	0.295	18	0.50
	T2D	8	0.043	0.05375	0.115	0.245	2.7	

T2D, type-2 diabetes; MBzP, monobenzyl phthalate; MCPP, mono(3-carboxypropyl) phthalate; MECPP, mono(2-ethyl-5-carboxypentyl) phthalate; MEHHP, mono(2-ethyl-5-hydroxyhexyl) phthalate; MEHP, mono(2-ethylhexyl) phthalate; MEOHP, mono(2-ethyl-5-oxohexyl) phthalate; MEP, monoethyl phthalate; MiBP, mono-iso-butyl phthalate; MMP, monomethyl phthalate; MnBP, mono-*n*-butyl phthalate; BPA, bisphenol A; BPS, bisphenol S; Limits of detection (LOD) (ng/mL) [MBzP = 0.06; MCPP = 0.028; MECPP = 0.24; MEHHP =0.17; MEHP = 0.4; MEOHP = 0.068; MEP = 1; MiBP = 0.63; MnBP =0.37; MMP = 0.23; BPA = 0.06; BPS = 0.11].

**Table 3:** Spearman’s rank order correlations of plasticisers and age, BMI and HbA1c in healthy controls and T2D.

Plasticiser	Controls						T2D					
	Age		BMI		HbA1c		Age		BMI		HbA1c	
	ρ	*p*-Value	ρ	*p*-Value	ρ	*p*-Value	ρ	*p*-Value	ρ	*p*-Value	ρ	*p*-Value
MBzP	0.348	0.04	0.253	0.16	0.275	0.13	0.413	0.03	−0.453	0.02	0.250	0.23
MCPP	0.018	0.89	0.018	0.89	0.137	0.27	0.122	0.48	0.361	0.03	−0.164	0.36
MECPP	0.045	0.69	−0.090	0.41	0.198	0.06	0.146	0.27	0.284	0.03	0.183	0.17
MEHHP	0.277	0.01	−0.013	0.91	0.047	0.68	−0.339	0.01	0.150	0.29	0.108	0.46
MEHP	0.092	0.42	−0.150	0.18	−0.150	0.17	−0.238	0.07	0.227	0.08	0.052	0.69
MEOHP	0.307	0.01	0.109	0.36	0.057	0.63	−0.088	0.59	−0.006	0.97	0.334	0.06
MEP	−0.126	0.25	−0.317	0.003	−0.235	0.03	−0.096	0.47	0.329	0.01	−0.042	0.75
MiBP	0.296	0.02	0.151	0.23	−0.068	0.57	−0.206	0.16	0.046	0.76	0.126	0.39
MnBP	0.224	0.04	−0.086	0.43	−0.065	0.54	−0.384	0.002	0.091	0.49	0.031	0.82
MMP	−0.201	0.13	−0.155	0.24	−0.213	0.03	0.196	0.27	−0.329	0.06	0.330	0.07
BPA	−0.009	0.96	−0.065	0.72	−0.122	0.48	−0.109	0.57	−0.217	0.25	0.293	0.13
BPS	0.249	0.047	0.051	0.69	0.016	0.89	−0.186	0.76	−0.559	0.33	0.725	0.17

T2D, type-2 diabetes; BMI, body mass index; HbA1c, glycosylated haemoglobin A1c; ρ, Spearman’s coefficient; *p*, statistical significance; MBzP, monobenzyl phthalate; MCPP, mono(3-carboxypropyl) phthalate; MECPP, mono(2-ethyl-5-carboxypentyl) phthalate; MEHHP, mono(2-ethyl-5-hydroxyhexyl) phthalate; MEHP, mono(2-ethylhexyl) phthalate; MEOHP, mono(2-ethyl-5-oxohexyl) phthalate; MEP, monoethyl phthalate; MiBP, mono-iso-butyl phthalate; MMP, monomethyl phthalate; MnBP, mono-*n*-butyl phthalate; BPA, bisphenol A; BPS, bisphenol S.

**Table 4:** Generalised linear models (GLMs) examining effects of group (T2D vs. healthy control), age and BMI on the levels of plasticisers.

Plasticiser	Predictor	B	SE	95% CI (Lower, Upper)	*p*-Value
MBzP	(Intercept)	−1.09	1.58	−4.20, 2.02	0.49
	Group 1	−0.89	1.98	−4.78, 2.98	0.65
	Group 2 (Ref)				
	Age	0.036	0.02	−0.01, 0.08	0.15
	BMI	−0.01	0.08	−0.18, 0.16	0.9
	Group (1) × Age	−0.003	0.02	−0.06, 0.05	0.91
	Group (1) × BMI	0.004	0.09	−0.17, 0.18	0.96
MCPP	Intercept	0.04	0.96	[−1.85, 1.93]	0.97
	Group 1	−3.41	1.42	[−6.20, −0.61]	0.01
	Group 2 (Ref)				
	Age	0.01	0.01	[−0.01, 0.04]	0.3
	BMI	0	0.04	[−0.09, 0.08]	0.93
	Group (1) × Age	0.03	0.01	[−0.001, 0.07]	0.04
	Group (1) × BMI	0.04	0.05	[−0.05, 0.14]	0.37
MECPP	(Intercept)	4.93	0.78	[3.39, 6.47]	<0.001
	Group 1	−5.28	1.24	[−7.72, −2.84]	<0.001
	Group 2 (Ref)				
	Age	0.004	0.01	[−0.02, 0.02]	0.74
	BMI	−0.1	0.03	[−0.17, −0.03]	0.01
	Group (1) × Age	0.01	0.01	[−0.01, 0.04]	0.28
	Group (1) × BMI	0.14	0.04	[0.06, 0.22]	<0.001
MEHHP	(Intercept)	3.07	1.1	[0.90, 5.24]	0.01
	Group 1	1.01	1.68	[−2.29, 4.31]	0.54
	Group 2 (Ref)				
	Age	0.02	0.01	[−0.002, 0.06]	0.07
	BMI	−0.08	0.05	[−0.18, 0.008]	0.07
	Group (1) × Age	−0.06	0.02	[−0.10, −0.02]	0.01
	Group (1) × BMI	0.07	0.05	[−0.02, 0.18]	0.14
MEHP	(Intercept)	2.88	1.02	[0.87, 4.88]	0.01
	Group 1	−0.4	1.42	[−3.20, 2.39]	0.77
	Group 2 (Ref)				
	Age	0	0.01	[−0.029, 0.02]	0.82
	BMI	−0.03	0.04	[−0.12, 0.05]	0.42
	Group (1) × Age	−0.02	0.01	[−0.05, 0.01]	0.23
	Group (1) × BMI	0.04	0.04	[−0.05, 0.13]	0.39
MEOHP	(Intercept)	1.57	1.23	[−0.84, 3.98]	0.2
	Group 1	1.45	1.86	[−2.19, 5.104]	0.43
	Group 2 (Ref)				
	Age	0.02	0.01	[−0.010, 0.060]	0.16
	BMI	−0.04	0.05	[−0.157, 0.066]	0.42
	Group (1) × Age	−0.05	0.02	[−0.099, −0.001]	0.05
	Group (1) × BMI	0.03	0.06	[−0.082, 0.153]	0.55
MEP	(Intercept)	10.69	1.16	[8.40, 12.98]	<0.001
	Group 1	−8.94	1.79	[−12.46, −5.43]	<0.001
	Group 2 (Ref)				
	Age	0.04	0.01	[0.00, 0.08]	0.01
	BMI	−0.26	0.04	[−0.35, −0.16]	<0.001
	Group (1) × Age	−0.02	0.02	[−0.06, 0.03]	0.48
	Group (1) × BMI	0.32	0.05	[0.22, 0.43]	<0.001
MiBP	(Intercept)	3.97	1.09	[1.83, 6.10]	<0.001
	Group 1	−0.72	1.61	[−3.89, 2.44]	0.66
	Group 2 (Ref)				
	Age	0.03	0.02	[−0.00, 0.06]	0.07
	BMI	−0.09	0.05	[−0.19, 0.01]	0.08
	Group (1) × Age	−0.04	0.02	[−0.08, 0.00]	0.08
	Group (1) × BMI	0.08	0.05	[−0.02, 0.19]	0.12
MnBP	(Intercept)	4.53	1.29	[2.00, 7.07]	<0.001
	Group 1	0.11	1.89	[−3.60, 3.81]	0.95
	Group 2 (Ref)				
	Age	0.04	0.02	[0.00, 0.07]	0.03
	BMI	−0.09	0.06	[−0.20, 0.02]	0.11
	Group (1) × Age	−0.06	0.02	[−0.10, −0.01]	0.01
	Group (1) × BMI	0.09	0.06	[−0.03, 0.20]	0.14
MMP	(Intercept)	3.3	1.01	[1.32, 5.29]	0.001
	Group 1	0.53	1.66	[−2.74, 3.79]	0.75
	Group 2 (Ref)				
	Age	−0.01	0.02	[−0.04, 0.03]	0.71
	BMI	−0.06	0.04	[−0.15, 0.02]	0.15
	Group (1) × Age	0.01	0.02	[−0.03, 0.06]	0.57
	Group (1) × BMI	0.01	0.05	[−0.09, 0.10]	0.88
BPA	(Intercept)	2.05	1.33	[−0.55, 4.65]	0.12
	Group 1	−0.26	1.62	[−3.43, 2.90]	0.87
	Group 2 (Ref)				
	Age	0.01	0.01	[−0.02, 0.04]	0.66
	BMI	−0.05	0.06	[−0.17, 0.06]	0.36
	Group (1) × Age	−0.02	0.02	[−0.06, 0.02]	0.39
	Group (1) × BMI	0.04	0.06	[−0.08, 0.16]	0.56
BPS	(Intercept)	5.81	1.65	[2.57, 9.04]	<0.001
	Group 1	−6.09	2.23	[−10.46, −1.71]	0.01
	Group 2 (Ref)				
	Age	0.08	0.02	[0.03, 0.12]	<0.001
	BMI	−0.36	0.06	[−0.48, −0.24]	<0.001
	Group (1) × Age	−0.09	0.03	[−0.14, −0.03]	0.003
	Group (1) × BMI	0.34	0.07	[0.21, 0.48]	<0.001

Group 1, T2D, type-2 diabetes; Group 2, healthy control; B, unstandardised regression coefficients; CI, confidence interval; BMI, body mass index; MBzP, monobenzyl phthalate; MCPP, mono(3-carboxypropyl) phthalate; MECPP, mono(2-ethyl-5-carboxypentyl) phthalate; MEHHP, mono(2-ethyl-5-hydroxyhexyl) phthalate; MEHP, mono(2-ethylhexyl) phthalate; MEOHP, mono(2-ethyl-5-oxohexyl) phthalate; MEP, monoethyl phthalate; MiBP, mono-iso-butyl phthalate; MMP, monomethyl phthalate; MnBP, mono-*n*-butyl phthalate; BPA, bisphenol A; BPS, bisphenol S.

## Data Availability

The raw data supporting the conclusions of this article will be made available by the authors on request.

## Abbreviations

The following abbreviations are used in this manuscript:

T2D	type-2 diabetes
BMI	body mass index
HbA1c	glycosylated haemoglobin A1c
DEHP	di-2-ethylhexylphthalate
DINP	di-isononylphthalate
LC	liquid chromatography
NHR	nuclear hormone receptors
PPAR	perturbed peroxisome proliferator-activated receptors
HPLC	high-performance liquid chromatography
SG	specific gravity
LC-MS/MS	liquid chromatography tandem mass spectrometer
sMRM	scheduled multiple reaction monitoring
SD	standard deviation
WHO	World Health Organisation
OGTT	oral glucose tolerance test
MEP	monoethyl phthalate
MnBP	mono-*n*-butyl phthalate
MCPP	mono(3-carboxypropyl) phthalate
MEHP	mono(2-ethylhexyl) phthalate
MECPP	mono(2-ethyl-5-carboxypentyl) phthalate
MEHHP	mono(2-ethyl-5-hydroxyhexyl) phthalate
MEOHP	mono(2-ethyl-5-oxohexyl) phthalate
MBzP	monobenzyl phthalate
MiBP	mono-iso-butyl phthalate
MMP	monomethyl phthalate
MiNP	mono-iso-nonyl phthalate
MnOP	mono-*n*-octyl phthalate
MCHP	monocyclohexyl phthalate
BPA	bisphenol A
BP-AF	bisphenol AF
BP-AP	bisphenol AP
BPB	bisphenol B
BPF	bisphenol F
BPS	bisphenol S
BPZ	bisphenol Z
DiBP	di-iso-butyl phthalate
